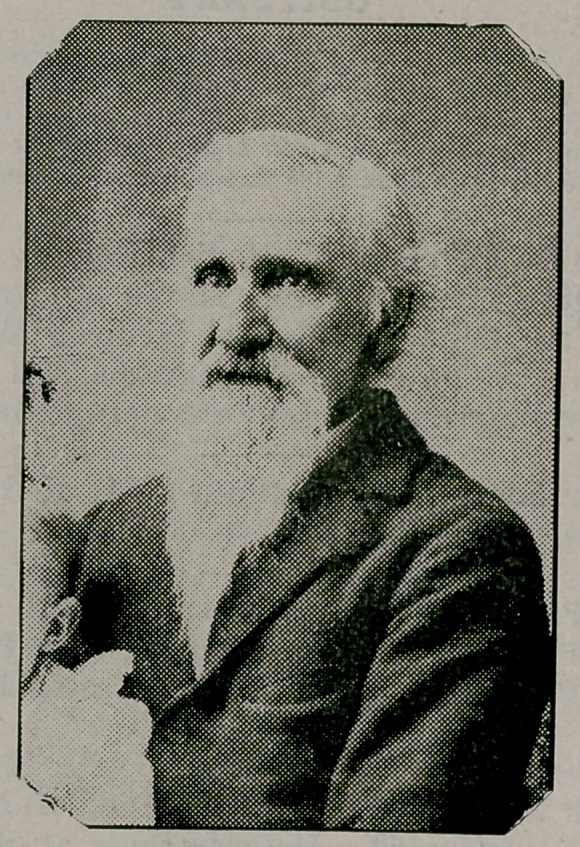# Dr. Jesse P. Bixby

**Published:** 1914-05

**Authors:** 


**Dr. Jesse P. Bixby,** Castleton Medical College (extinct 1861) 1852, died at his home in Rushford, April 3. He was born in Mt. Holley, Vt., Dec. 27, 1821, and after attending the Black Creek Academy, studied medicine at the Woodstock Medical

College before going to the Castleton College. His entire professional life was spent at Rushford, except that he practiced at Farmersville Station from 1902 to 1904. He was a prominent Mason.

## Figures and Tables

**Figure f1:**